# Interaction between path integration and visual orientation during the homing run of fiddler crabs

**DOI:** 10.1098/rsos.170954

**Published:** 2017-09-20

**Authors:** Hisashi Murakami, Takenori Tomaru, Yukio-Pegio Gunji

**Affiliations:** 1 Department of Information Systems Creation, Kanagawa University, Yokohama, Kanagawa, Japan; 2 Department of Computer Science and Engineering, Toyohashi University of Technology, Toyohashi, Aichi, Japan; 3 School of Fundamental Science and Engineering, Waseda University, Shinjuku, Tokyo, Japan

**Keywords:** path integration, visual cue, homing behaviour, fiddler crab, *Uca*

## Abstract

Foraging fiddler crabs form a strict spatial relationship between their current positions and burrows, allowing them to run directly back to their burrows when startled even without visual contacts. Path integration (PI), the underlying mechanism, is a universal navigation strategy through which animals continuously integrate directions and distances of their movements. However, we report that fiddler crabs also use visual orientation during homing runs using burrow entrances as cues, with the prioritised mechanism (i.e. PI or visual) determined by the distance (which has a threshold value) between the goal, indicated by PI, and the visual cue. When we imposed homing errors using fake entrances (visual cue) and masking their true burrows (goal of PI), we found that frightened fiddler crabs initially ran towards the true burrow following PI, then altered their behaviour depending on the distance between the fake entrance and masked true burrow: if the distance was large, they kept running until they reached the true burrow, ignoring the visual cue; however, if the distance was small, they altered the homing path and ran until they reached the fake entrance. This suggests that PI and visual mechanism in fiddler crabs are mutually mediated to achieve their homing behaviour.

## 1. Introduction

Most animals, including humans, use a navigational strategy known as path integration (PI) [1–4]. Through PI, animals continuously sum the directions and distances of their movements to form a single vector (home vector) that links their current location with a significant starting point, such as a nest or burrow. Previously, it was considered that although animals use PI as a primary strategy in unfamiliar environments or vast, featureless areas, such as desert ants foraging in the Saharan salt pans, they usually use it as a backup strategy to other navigational strategies (e.g. visual orientation by landmarks and olfactory cues) in familiar circumstances [5–8]. Recent experiments on insect navigation, however, emphasize that such guidance systems tend to combine with the control direction in a weighted manner [9–13].

Most fiddler crabs (*Uca* spp.) are short-range, central-place foragers, with males in particular using their burrows as a hub for foraging or attracting or visiting females in the neighbourhood. These crabs rely almost exclusively on PI to return to their burrows [14–20] with exceptions mentioned later [21–23]. The crabs leave their burrows to forage on the tidal flats and, if frightened, scuttle directly back to their own burrows, even when they are closer to the burrows of other crabs [14,19]. Although foraging crabs generally cannot see the entrance to their burrow owing to perspective foreshortening [16,17,21], the crabs continuously turn their body axis towards their burrow allowing them to return rapidly and precisely, even though the entrance of the burrow is masked [14,18]. In this way, fiddler crabs form a strict spatial relationship between their current position and home via PI.

Passive translation experiments have been conducted to investigate the PI system of fiddler crabs. While the visual system of fiddler crabs is exquisitely tuned to the geometry of vision in the flat world they live in and is sensitive to the rotational optic flow that is induced by passive rotation [14,19], crabs that are actively feeding do not compensate for passive translocation. Thus, when a foraging crab is shifted, for instance, on a sliding sheet of sand paper, by following a straight path it subsequently returns to a location that corresponds to where the burrow would have been had the translocation not taken place, irrespective of the presence of landmarks near to its burrow [14]. This suggests that the visual mechanism basically does not function during rapid escape until use of the home vector has been exhausted [18]. It has also been shown that stride integration, as occurs in desert ants [2,3], is used for precise distance measurement in fiddler crabs [20,24], rather than optic flow as in honeybees [4] or linear acceleration as in humans.

However, the PI system can rapidly accumulate errors in the absence of external cues [25–27] and indeed homing errors of a few centimetres have been reported in fiddler crabs during translocation experiments [14,19,24]. Recent studies have reported that active territorial behaviour or courtship behaviour can lead to such homing errors, and that some species of fiddler crabs can compensate for this by using the courtship structure (known as a semidome, which was originally considered to be built at the burrow entrance for courtship during the breeding season [28–30]) as a landmark before undertaking the homing run by PI (i.e. to update the home vector) or after it (i.e. searching after the run) [21–23]. This suggests that fiddler crabs may use the burrow entrance as a visual cue to compensate for the usual homing error when no semidome is present. It is not known when (before, after or during the homing run) they compensate for such error, but the predominance of their PI system and their perspective foreshortening would suggest that they do so after the straight homing run (i.e. after exhausting the home vector that was formed while foraging).

In this study, we aimed to determine when and how fiddler crabs compensate for homing errors. We first conducted a preliminary experiment to determine whether fiddler crabs use their burrow entrances as visual cues during an escape run. To do this, we superposed a fake entrance (a hole at the centre of a square cork sheet) on the true burrow entrance when the crab was inside its burrow and then moved the sheet once the crab had emerged from this superposed burrow, allowing us to create a situation where the fake entrance was shown while the true entrance was masked by the sheet. If the PI of fiddler crabs is strict and any error is compensated for after the straight homing path has been completed, we would expect frightened fiddler crabs to first arrive at the location of the masked true entrance and then search for the burrow. However, we found that they actually ran back towards the fake entrance without arriving at the location of the true burrow first, indicating that they could compensate for their homing errors by using burrow entrances as visual cues. Therefore, this raised the question of whether this compensation occurred before or during the homing runs, as well as over what distance the crabs can compensate for such errors.

Therefore, we next conducted an experiment in which the fake entrance was shown and the true entrance was masked by an oblong structure, and the distance over which the oblong sheet was translocated was varied. With video analysis, we first evaluated the threshold value of the translocation distance, up to which the crabs can compensate for the mismatch between their true burrow location and the fake burrow entrance. We next determined whether the crabs compensate before or during the run when they move to the fake entrance: if they compensate for any error before starting their escape runs, they will follow a straight homing path and directly arrive at the fake entrance, and if they compensate for the errors in the middle of their homing runs, they will initially orient towards the true burrow at the starting point of the run but will finally run to the fake entrance without arriving at the location of the true burrow.

## 2. Material and methods

Field experiments were conducted in a large colony (approx. 15 × 20 m) of *Uca perplexa* (H. Milne Edwards, 1852) at Funaura Bay on Iriomote Island, Okinawa Prefecture, Japan (123°48′ E; 24°24′ N) during the day from 1.5 h before to 1.5 h after low tide in August 2016. We used male *U*. *perplexa* that show typical homing behaviour using PI. All experiments were recorded from above using a Panasonic HDC-TM700 camcorder (1920 × 1080 pixels, 30 frames s^−1^) fitted with a Panasonic VW-W4907H-K wide conversion lens (0.75×) on a four-legged steel frame resulting in a recording area of 3 × 1.7 m. Immediately after the camera was set up, each experiment trial was conducted with a randomly chosen individual in the area.

### 2.1. Preliminary experiment with translocation of a fake entrance on a square sheet

We first tested whether the crabs responded to entrances as visual cues. A fake entrance was constructed by cutting a 1.5 cm diameter hole in the centre of a 10 × 10 cm cork sheet, to which a plastic sheet of the same size was attached for reinforcement (2 mm thickness in total). This fake entrance was then superposed on the true burrow entrance when the crab was inside its burrow. We observed that crabs emerged from this modified burrow entrance, foraged across the terrain, engaged in homing behaviour and defended the burrow from other intruding crabs, which is similar to behaviours that are observed under natural conditions.

Two corners of the sheet were attached to a fishing line, allowing an experimenter, who was sitting on a chair 1.5 m away, to pull the sheet towards him. After the crab had emerged from its burrow, left the fake entrance and the sheet and moved away to feed, the sheet was translocated to allow either the fake entrance to be shown and the true entrance to be masked by the sheet; or the true entrance to be shown in addition by translocating the sheet a greater distance. The translocation of the sheet was performed slowly so as not to disturb the crab. Then the crab was frightened by the experimenter getting up from the chair. The latter situation was used to test whether the crabs responded to the fake entrance that visually corresponded to the entrance from which they had emerged but was not located at the end of the home vector they had formed during foraging, even though the true entrance, which visually differed from the entrance from which they had emerged, was located at the end of the vector. In total, 15 trials were conducted for each of the above conditions.

### 2.2. Experiments with translocation of a fake entrance on an oblong sheet

**Figure 1.**
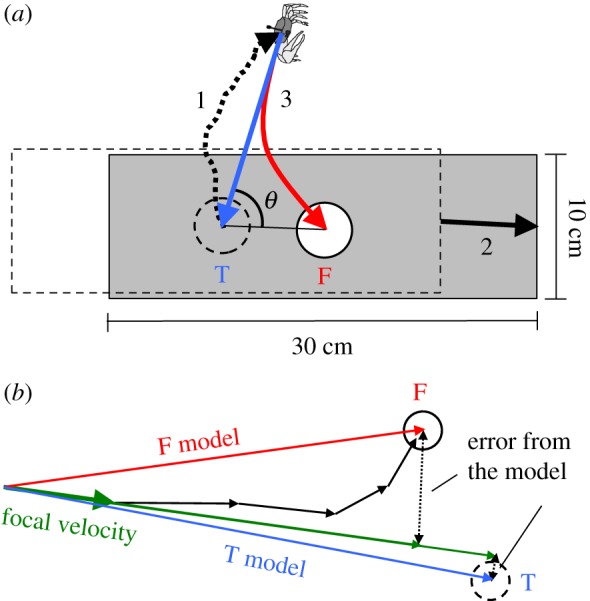
Experimental set-up and measurement procedure. (*a*) After a crab had emerged from its own true burrow (dashed circle labelled ‘T’ in blue), which was superposed by a fake entrance (solid circle labelled ‘F’ in red) cut into the centre of a cork sheet and had moved away from the sheet (1), the sheet was translocated so that the true burrow was covered and only the fake entrance was shown (2). We then observed whether the crab ran back to the true burrow or fake entrance when frightened (3; red or blue arrow, respectively). Moreover, to check the geometrical relation among the crab's starting point and both entrances, we calculated the relative angle between the direction of translocation and the direction from the crab's starting point to the true entrance (solid arc labelled ‘*θ*’), which corresponds as in figure 2*b*. (*b*) Schematic diagram showing how errors from the two direct path models were calculated. The F model (red arrow; direct path to fake entrance) and T model (blue arrow; direct path to true burrow) were calculated at each position during an escape run, and errors from the models were then measured as distances between the end of the model and the end of a vector (green thin arrow) whose direction corresponded to the crab's velocity at a particular position (green thick arrow labelled ‘focal velocity’) and whose length was scaled with the model.

Based on observations from the preliminary experiment (see Results), we conducted experiments using a fake entrance on an oblong sheet (10 × 30 cm) to determine when (before or during homing runs) the crabs were compensating for the mismatch between their true burrow location and the fake burrow entrance and how long they could do so for. These experiments were carried out in the same way as the preliminary experiment, except that the distance over which the oblong sheet was translocated varied over a distance that still allowed the true entrance to be masked (figure 1*a*). In total, 40 trials were conducted for this experiment.

### 2.3. Video and data analysis

Time series of the crabs' positions in all trials of the main experiments using the oblong sheet were tracked frame by frame (30 frames s^−1^) from video images using image-processing software (Library Move-tr/2D v. 8.31; Library Co. Ltd., Tokyo, Japan) as a single pixel whose side length was 1.58 mm. We considered the starting point of an escape run as a crab's position at a frame after being frightened and when it moved further than 3.33 mm in the 33.3 ms time interval preceding this frame and further than 10.0 mm over a three-frame interval (100 ms) starting at the previous frame, and the stopping point as a crab's position at a frame after the starting point and when it moved less than 3.33 mm in the 33.3 ms time interval preceding this frame and less than 10.0 mm over a three-frame interval starting at the previous frame. Note that during escape runs, crabs in 5 out of 40 trials were caught by the edge of the sheet and the stopping points were located here; therefore, we excluded these trials from the analysis. Where the stopping point was closer to the fake entrance than the location of the masked true entrance, we considered that the crab had run back to the fake entrance and called these trials ‘F events’. Conversely, when the stopping point was closer to the masked true entrance than the location of the fake entrance, we considered that the crab had moved to the true burrow and called these trials ‘T events’. To check the geometrical relation among the crab's starting point and both entrances, we calculated the relative angle between the direction of translocation (i.e. the direction from the true entrance to the fake entrance) and the direction from the crab's starting point to the true entrance (figure 1*a*).

We fitted a multiple logistic regression model to evaluate whether changes in the probability of occurrence of an F event could be explained by the translocation distance, including the distances to the true burrow and the fake entrance from the starting point as factors. To determine the distance between the fake and true entrance over which the fiddler crabs could compensate, we then fitted a simple logistic regression to evaluate the relationship between the probability of an F event and the translocation distance.

To determine whether the crabs oriented towards the fake entrance or true burrow at the starting point, we used two direct path models (figure 1*b*). The first model, F, was considered as a vector linking the starting point with the location of the fake entrance, i.e. a direct path to the fake entrance (which would be consistent with the situation where a home vector was updated before the crab was frightened). The second model, T, was considered as a direct path to the true burrow (which would be consistent with the crab using the home vector formed during foraging). For each model, we defined an initial error as the distance between the end of the model (entrance location) and the end of a vector whose direction corresponded to the crab's velocity at the starting point and whose length was scaled with the model. Thus, if a crab oriented towards the fake entrance from the beginning of a run, the error from the F model would be smaller than that from the T model; whereas if a crab initially oriented towards the true burrow (even though it may have finally arrived at the fake entrance in an F event), the error from the T model would be smaller than that from the F model. Therefore, we considered that the model was plausible for the initial direct path model if it had the smallest error value and assumed that the direction of the model represented the crab's initial orientation.

We also calculated direct path models and errors associated with them at each of a crab's positions during an escape run to observe whether and how the errors in both direct path models changed through time and compared the final errors (i.e. the error at the stopping point) between the models to visualize the transitions in them using an averaged error versus time curve. Since the escape runs of both events occurred over different lengths of time, they each had different numbers of data points. Therefore, we used an average curve to reflect the shape of the individual curves, not necessarily the precise timing. To calculate the averaged error versus time curve, individual curves were normalized to their maximum time (i.e. all curves were given a relative time axis ranging from 0 to 1), as performed by Layne *et al*. [19]. The data were then graphically redigitized at an arbitrarily high spatial frequency so that all curves had 216 data points, following which the error at each point was averaged. All statistical analyses were performed with R v. 3.1.2.

## 3. Results

### 3.1. Use of entrances as visual cues

In the preliminary experiment, the frightened crabs ran rapidly and abruptly stopped running at the location of the fake entrance or the true burrow or entered the true entrance if it was visible. We found that when the true entrances were visible, all crabs ran to the true burrows. However, when the true entrances were masked, most crabs ran to the fake entrances (frequency of runs to fake entrance; true entrance shown versus true entrance masked: 0/15 versus 13/15; *χ*^2^ test: *χ*_1_^2^ = 19.5, *p* < 0.0001). Therefore, if an entrance was located at the end of a crab's home vector that was formed during foraging, the crab moved there depending almost entirely on the home vector and ignored the fake entrance. However, if there was a mismatch between the location of an entrance and the end of the home vector, the crab compensated for this either before or in the middle of its homing run in this preliminary experiment.

### 3.2. Burrow selection

**Figure 2.**
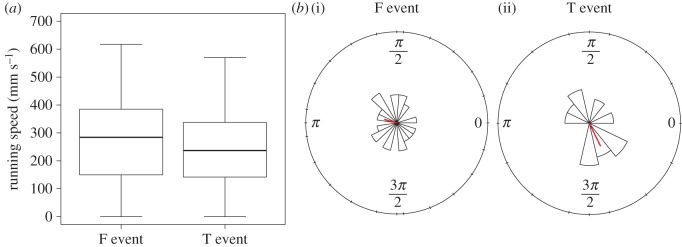
(*a*) Running speed of fiddler crabs (*Uca perplexa*) during the escape run in F events (returned to fake entrance) and T events (returned to true burrow). Boxes represent the interquartile ranges with the median line. (*b*) The rose diagram of the direction of the translocation relative to the line connecting the position of the true entrance and the crab's starting point in the F (i) and T events (ii). Red lines indicate the direction and magnitude of the mean resultant vector.

**Figure 3.**
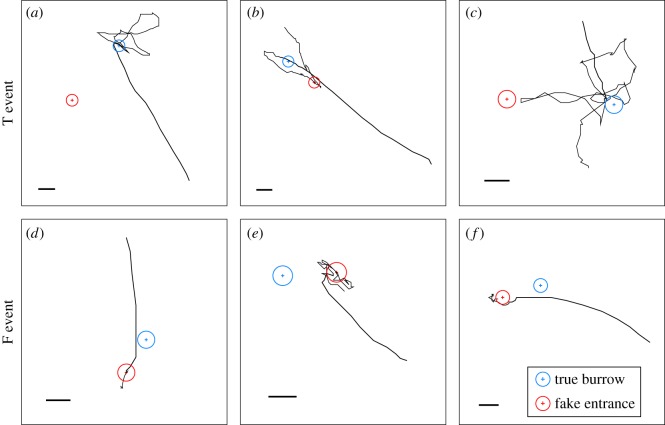
Examples of reconstructed trajectories for a T event (returned to true burrow; *a*–*c*) and an F event (returned to fake entrance; *d*–*f*). Thick and thin lines show the trajectories during and after the escape run, respectively. Red and blue circles represent the locations of the fake entrance and true burrow, respectively. Scale bars, 20 mm.

As observed in the preliminary experiment, in the main experiments, the frightened crabs ran rapidly and stopped running abruptly at either the location of the fake entrance or that of the masked true entrance. In total, we observed 14 T events and 21 F events. In the T events, the crabs ran in a straight line and stopped running at the location of the masked true entrance. Some crabs got close to the fake entrance for a few seconds or after a stereotypical crisscross search, while the others continued to search. In the F events, crabs ran towards the fake entrance without stopping at the location of the masked true entrance. Some crabs repeatedly attempted to enter the fake entrance regardless of the absence of a burrow and then searched in its vicinity. It should be noted that crabs in both events ran without stopping until they arrived at either entrance, and that there is significant but slight difference in running speeds during escape runs between the two events (Wilcoxon's rank-sum test: *W* = 47945, *p* = 0.004379, *n *= 593; figure 2*a*) that is unexplained but may relate to whether crabs ran, finally having visual contact with the entrance during the runs. The translocation direction of the fake entrance relative to the direction from the crab's starting point to the true entrance did not differ significantly between both trials (Watson−Wheeler test: *W *= 0.358, *p *= 0.836, *n *= 35; figure 2*b*). Examples of the escape runs in T events and F events are shown in figure 3.

### 3.3. Maximum translocation distance for escape runs to fake entrances

**Figure 4.**
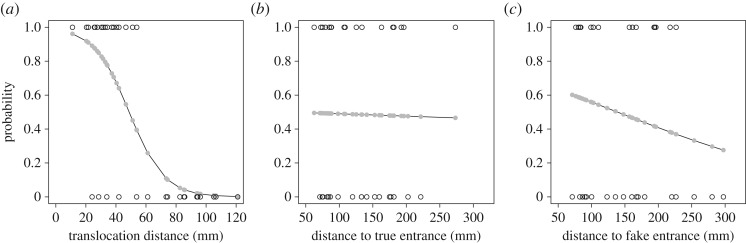
Probability of the occurrence of an F event in relation to the translocation distance of the sheet (*a*) and the distance to the true (*b*) and the fake entrances (*c*). Open circles represent an F event (returned to fake entrance; 1) or T event (returned to true burrow; 0). The line shows the fitted logistic regression with expected values (grey filled circles).

The distances to the fake entrance and the true burrow did not significantly affect the probability of occurrence of an F event, whereas the translocation distance did (table 1). Therefore, we plotted F events (1) and T events (0) against translocation distance and conducted a logistic regression (figure 4*a*). The maximum translocation distance at which F events were observed was 53.8 mm. The distance at which F events had a 0.5 probability of occurrence was estimated to be 48.8 mm, while the probability that an F event would occur beyond 120 mm was estimated to be 0.0023. We also plotted F events and T events against the distances to the fake entrance and the true burrow and conducted a logistic regression (figure 4*b*,*c*).

**Table 1.** Logistic regression analyses evaluating how the probability of the occurrence of an F event (crab returned to fake entrance) is affected by the translocation distance, distance to the true burrow and distance to the fake entrance (top, multiple regression) and each factor (below, simple regression). CI, confidence interval.

factor	*β* estimates	*p*-value	95% CI
multiple regression			
intercept	3.01	0.0389	0.413 to 6.35
translocation distance	−0.0777	0.00877	−0.149 to −0.0310
distance to true burrow	0.00580	0.760	−0.0324 to 0.0448
distance to fake entrance	−0.00307	0.860	−0.0402 to 0.0319
simple regression with translocation distance			
intercept	4.16	0.00230	1.23 to 6.06
translocation distance	−0.0853	0.00362	−0.141 to −0.0321
simple regression with distance to true entrance			
intercept	0.0156	0.986	−1.93 to 1.67
distance to true entrance	−0.000556	0.932	−0.0149 to 0.0119
simple regression with distance to fake entrance			
intercept	0.845	0.348	−1.10 to 2.47
distance to fake entrance	−0.00608	0.282	−0.0190 to 0.00466

### 3.4. Compensation for errors during rapid escape runs

**Figure 5.**
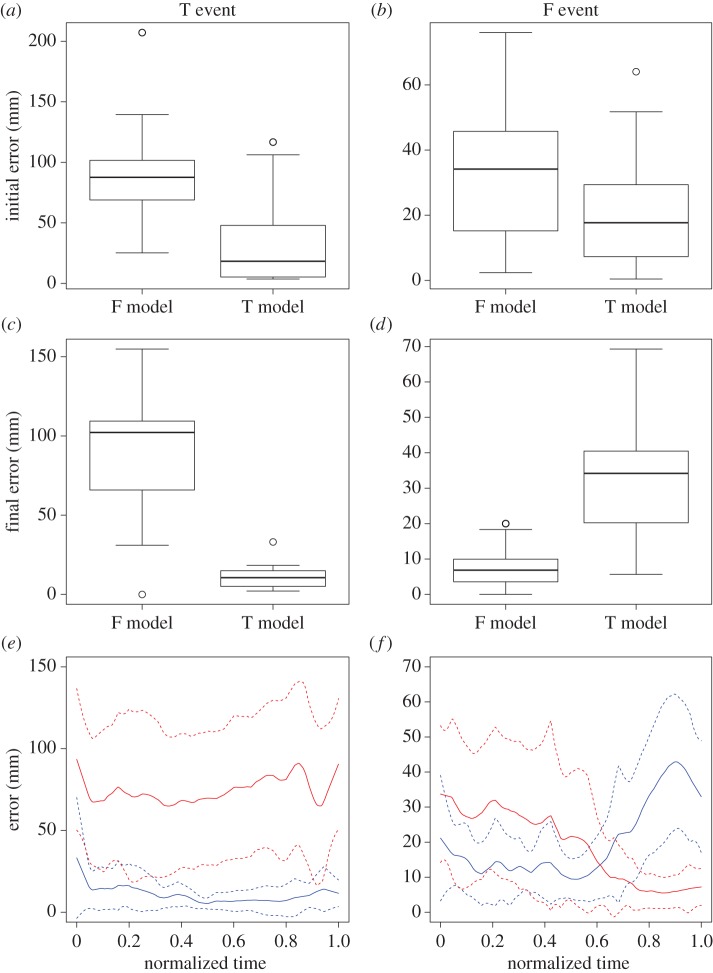
Error from each model in a T event (returned to true burrow; left) and F event (returned to fake entrance; right). (*a*,*b*) Initial error; (*c*,*d*) final error. Boxes represent the interquartile ranges with the median line. (*e*,*f*) Each error during the escape run plotted against normalized time. Red line, error from the F model; blue line, error from the T model. Horizontal lines show the mean error ± standard deviation (dotted lines).

During a T event, the crab's initial error from the T model was smaller than that from the F model (Wilcoxon's rank-sum test: *W* = 170, *p* = 0.000523, *n* = 28; figure 5*a*), and the same was true for the final error (Wilcoxon's rank-sum test: *W* = 181, *p* = 3.4 × 10^−5^, *n* = 28; figure 5*c*). Furthermore, the averaged error from the T model versus time curve was always lower than that of the F model curve during escape runs (figure 5*e*). These findings indicate that crabs in the T event consistently ran along a direct path to the true burrow during escape runs. By contrast, during an F event, although the crab's initial error from the T model was once again smaller than that from the F model (Wilcoxon's rank-sum test: *W* = 307, *p* = 0.0295, *n* = 42; figure 5*b*), the reverse was true for the final error (Wilcoxon's rank-sum test: *W* = 225, *p* = 3.74 × 10^−7^, *n* = 42; figure 5*d*). Furthermore, the averaged error from the T model versus time curve gradually became larger while that of the F model curve gradually became smaller, with the two intersecting in the middle of the escape run (figure 5*f*). These findings indicate that while crabs in the F event initially oriented to the location of the true burrow, they subsequently modified their homing path in the middle of the escape run to finally arrive at the fake entrance, depending on the translocation distance.

**Figure 6.**
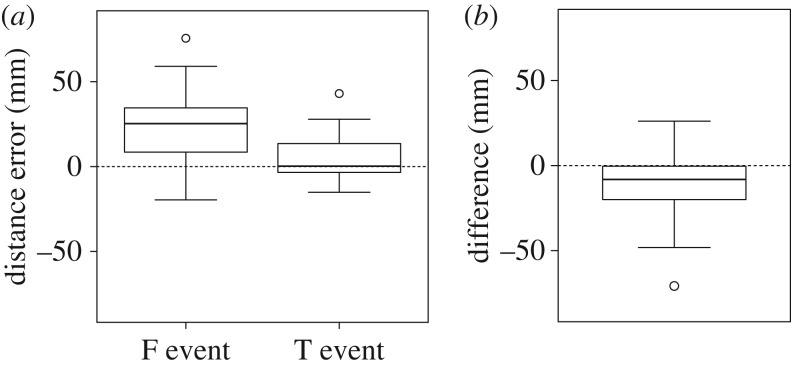
Evaluation of the total path length. (*a*) Distance error in F events (returned to fake entrance) and T events (returned to true burrow) calculated as the difference between the total path length during an escape run and the distance between the starting point and the true burrow. (*b*) Difference between the total path length and the sum of the length of a direct path to the true burrow and the distance between the true burrow and the fake entrance in F events. Boxes represent the interquartile ranges with the median line. The dashed line is the zero distance error or difference.

We then calculated the difference between the total path length during an escape run and the length of a direct path to the true burrow (i.e. the distance between the starting point and the true burrow) as a distance error (figure 6*a*). A comparison of the absolute error values showed that the error value in an F event was larger than that in a T event (Wilcoxon's rank-sum test: *W* = 237, *p* = 0.00185, *n* = 35), indicating that if the crab detected an error in PI by a visual cue (entrance) during the escape run, it no longer considered the length of the home vector calculated during foraging. Moreover, a comparison of the total path length in an F event with the sum of the length of a direct path to the true burrow and the distance between the true burrow and the fake entrance (i.e. the length of the shortest path to the fake entrance via the location of the true burrow) showed a significant difference, whereby we also confirmed that the crab ran to the fake entrance without going through the location of the true burrow (Wilcoxon's one-sample test: *V* = 56, *p* = 0.0384, *n* = 21; figure 6*b*).

## 4. Discussion

Fiddler crabs use path integration to locate their burrows from distances at which they cannot see the burrow. When disturbed, fiddler crabs ‘play out’ the home vector and stop when it is exhausted, allowing them to rapidly and accurately return to their burrows via their predominant PI system. In this study, we examined when and how fiddler crabs compensate for an error that would inevitably occur at some stage during foraging. To investigate this, we imposed such an error by showing the crabs a fake entrance and masking the true entrance, i.e. a fake entrance was employed as a visual cue. Because we did not interfere with the crabs per se, they should have directly run to the location of the true burrow if escape runs are exclusively performed by the home vectors calculated during foraging. However, we observed that the crabs sometimes ran towards the fake entrance without stopping at the true burrow. Moreover, they should have oriented towards the fake entrance from the beginning of the run if they perceive it as the goal of their path before the run. However, we found that they always oriented towards the true burrow at the beginning of the escape run, while they finally arrived at the fake entrance. Therefore, we conclude that fiddler crabs can compensate for their homing errors during runs. Moreover, our logistic regression analysis suggested that this compensation is restricted by the distance between the location of the visual cue (entrance) and the end of their home vector. Note that, in some trials, the crab's starting point and the fake entrance might be within its visual contact distance that is approximately five times the eye height above ground [21,31,32] and is roughly estimated between 4 and 8.5 cm for *U. perplexa*. Considering this, it should be noted again that there is a possibility that crabs in such trials perceived the fake entrance before being frightened. However, this would be unlikely for the following reasons; first, crabs that are actively feeding do not respond to the passive translocation, although the surroundings within the visual contact distance could be perceived to be moving; second, crabs in our experiment as well did not respond to the translocation of the platform; third, as we mentioned in the Results, our logistic regression analysis showed that even if the distance between a crab and the fake (or true) entrance at the starting point is within the visual contact distance it did not significantly affect the crab's burrow selection (figure 4*b*,*c*). Therefore, we conclude it unlikely that crabs perceive the fake entrance as the goal of their path before the run.

While crabs can measure the requisite distance by stride integration, we also observed that the total path length was changed if the error was detected using a visual cue. Such changes in distance during escape runs have previously been reported by Walls & Layne [24], who found that crabs which were passively translocated so that their home vector lay across difficult terrain such as a steep slope mound could calculate this vertical detour and accurately travel the correct horizontal distance, resulting in a longer path length than the direct path that would be expected on flat ground. Although there were no such topographic changes in our experiment, the total path length was still larger than when crabs ran directly to the location of their own burrows. Hence, we conclude that these changes were induced by the visual cues.

These findings indicate that the PI system can be suppressed by the visual system in fiddler crabs. Such an interaction between the PI and visual mechanisms has also been found in other animals. For instance, desert ants can give priority to information from a landmark cue rather than from PI if there is a conflict between the two during their long journey, but then continue to follow the path given by PI if no further cue appears [7]; and honeybees flying along a straight path can reach a feeder far from the starting point, but the accuracy of the distance they fly before searching for the feeder is improved when they pass a landmark and the distance is shortened by the amount that such a landmark is moved towards the start of the path [6]. Thus, such animals were considered to use PI as a backup strategy for navigation [5,7]. However, recent studies, in particular on desert ants, presented an alternative explanation that the PI system and landmark guidance (and/or other navigational strategies) could be in operation simultaneously [9–13]. In other words, there is a heterarchical network among different navigational systems, in which systems tend to combine to control the direction in a weighted manner, rather than a dominant hierarchy. For instance, desert ants' directional certainty in PI is the strongest when it is far from its goal and becomes less as distances shorten [11,12].

Regarding this point, our results suggest that the navigation system of a fiddler crab is also heterarchical, but whether it is performed in a weighted manner remains unclear. As mentioned previously, the escape runs of fiddler crabs depend almost exclusively on PI whether there is a landmark near to them or another crab's burrow at a closer distance [14]. Moreover, our results suggest that the burrow entrance is used as a visual cue only if it is near to the end of their home vector and, if not, the crabs perform the usual straight path to the location of the true burrow until their home vector is exhausted, ignoring the fake entrance. While further data are required to better understand this mechanism, it seems likely that even though visual cues may be available at a certain point along their home vector, visual orientation will be suppressed by PI until they approach the end of the vector. Then, at this stage, it remains to be seen whether the crabs solve conflicts between information provided by PI and the visual appearance of the burrow in a weighted manner, as it has been suggested for ants [9–13], or whether PI and visual orientation are mutually exclusive and are immediately shifted from one to the other only if crabs approach the end of the home vector and the burrow entrance becomes visible. However, in any case, PI and the visual system in fiddler crabs may be mutually mediated to achieve their characteristic rapid homing behaviour, rather than one acting as a dominant strategy or backup for the other. Then, this would be an example of a very fast and multi-modal decision-making process, which is of great interest, especially when considering its neural implementation.

## Supplementary Material

Supporting data for "Interaction between path integration and visual orientation during the homing run of fiddler crabs"

## Data accessibility

All data are available in the electronic supplementary material.
